# Spinal Cord Meningioma: A Treatable Cause of Paraplegia

**DOI:** 10.4021/jocmr912w

**Published:** 2012-07-20

**Authors:** Manoj K. Mittal, Alejandro A. Rabinstein

**Affiliations:** a Mayo Clinic, Rochester, MN, USA

**Keywords:** Spinal cord compression, Meningioma, Chondrocalcinosis

## Abstract

Chondrocalcinosis associated with Gitelman syndrome (GS) presents in young adults with either no symptoms or joint pain, muscle weakness, muscle cramps, paresthesias, episodes of tetany, or hypokalemic paralysis. Spinal cord meningiomas present with gradual onset of lower extremities weakness, numbness, pain, or balance problem. We report a 76 year old gentleman who presented with gradually progressive leg weakness puzzling the treating physicians.

## Introduction

Chondrocalcinosis associated with Gitelman syndrome presents in young adults with either no symptoms or joint pain, muscle weakness, muscle cramps, paresthesias, episodes of tetany, or hypokalemic paralysis [1]. Spinal cord meningiomas present with gradual onset of lower extremities weakness, numbness, pain, or balance problem. We report a 76 year old gentleman who presented with gradually progressive leg weakness, puzzling the treating physicians.

## Case Report

**Figure 1:**
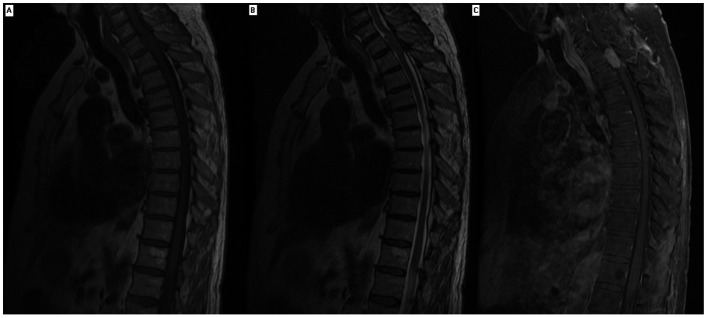
Magnetic resonance imaging of thoracic spine of a patient with paraplegia. Contrast enhancing magnetic resonance imaging showed solid homogenous intradural extramedullary mass extends from level of first thoracic vertebrae superior endplate to superior aspect of second thoracic vertebrae resulting in spinal cord compression. The mass was isointense on MRI T1 and T2 sequence.

Thirteen months ago a 76 year-old man with previous history of osteoarthritis presented to his family physician with gait instability, right foot drop, and falls. His symptoms were thought to be from osteoarthritis and he was referred to an orthopaedic surgeon who found some tenderness at his knees without any malalignment or effusion. Radiographs of his knees showed mild chondrocalcinosis. He was diagnosed with calcium pyrophosphate deposition disease and was treated with intraarticular corticosteroid injections without any improvement. A podiatrist noted high steppage gait eight months ago and recommended shoe orthotics and a walker. Three months ago the orthopedic surgeon noted bilateral quadriceps atrophy and thought it to be secondary to deconditioning from chondrocalcinosis. Patient saw his primary physician one month ago for inability to walk and difficulty transferring from bed to wheelchair, again thought to be from progressive chondrocalcinosis. Two weeks ago the patient went to urgent care for urinary tract infection. A different family physician saw him and requested a neurology consultation. Patient told the neurologist about progressive leg weakness, numbness and tingling ascending up to mid back over the last year and urinary retention over the last three weeks. Neurological examination revealed decreased pin prick sixth thoracic vertebra down, complete plegia on right leg, severe weakness in the left leg, and bilateral positive Babinski’s sign. Serum potassium was normal. Contrast enhanced spinal magnetic resonance imaging (MRI) suggested thoracic meningioma causing spinal cord compression (Fig. 1). Patient was referred to Mayo Clinic and underwent surgical resection of his tumor. Pathology came back positive for World Health Organization (WHO) grade 1 meningioma (meningothelial and psammomatous type). Two days after surgery motor strength had started to improve in both legs.

## Discussion

Our patient had slowly progressive paraplegia favoring the diagnosis of a slowly progressive spinal myelopathy caused by slowly growing tumors, which can be extraaxial (meningioma, neurofibroma, lipoma) or intraaxial (ependymoma, astrocytoma). Difficulty walking, motor, and sensory deficits are seen in more than 80% of spinal meningiomas [2]. Contrast enhanced MRI spine is the study of choice [3]. Median delay in diagnosis of intradural spinal tumors has been reported to be 2.5 years. Eighty percent of spinal tumor patients are initially referred to specialty other than neurology or neurosurgery [4]. Surgical intervention may result in independent walking in up to 80% patients at one year follow up [5].

Our patient had seen multiple physicians who noted the gradual progressive weakness in the lower legs and balance problems and thought it to be from osteoarthritis and chondrocalcinosis. The cause of leg weakness in our patient was not chondrocalcinosis as these patients are usually younger and does not have foot drop or muscle atrophy. Clinical symptoms like joint pain, muscle weakness, muscle cramps, and paresthesias may overlap between chondrocalcinosis and meningioma making it difficult to distinguish between the two. Spinal cord lesions usually present with a spinal sensory level, bladder and bowel dysfunction, hypertonia in lower extremities, upgoing plantar response, back pain with radiation to the hip, knee or toes, paresthesias, and weakness of both proximal and distal muscles. Presence of any of these findings should alert about a possible spinal cord lesion.

Delay in diagnosis of spinal cord diseases like meningioma may result in increased patient morbidity and may decrease the chances of good functional outcome. Primary care providers should consider early MRI spine and neurological evaluation in patients with new onset paresthesia, motor weakness, urinary incontinence or retention, frequent falls, and unexplained pain in lower back or legs.

## Abbreviations

MRI: magnetic resonance imaging
WHO: World Health Organization
